# Bilateral Upper Extremity Superficial Venous Thrombosis and Altered Mental Status as Initial Manifestations of Aggressive B‐Cell Lymphoma: A Case Report

**DOI:** 10.1002/ccr3.73341

**Published:** 2026-08-21

**Authors:** Milad Rezvani, Mahdi Naeiji, Hasan Maghsoudifar, Sepehr Zamani, Maryam Yarmohammadi, Maryam Valikhani

**Affiliations:** ^1^ Student Research Committee, School of Medicine Shahroud University of Medical Sciences Shahroud Iran; ^2^ Imam Hossein Center for Education, Research, and Treatment Shahroud University of Medical Sciences Shahroud Iran; ^3^ School of Medicine Shahroud University of Medical Sciences Shahroud Iran; ^4^ Clinical Research Development Unit, Imam Hossein Hospital Shahroud University of Medical Sciences Shahroud Iran

**Keywords:** B‐cell lymphoma, CD20‐positive, confusional state, fever of unknown origin, venous thromboses

## Abstract

A 72‐year‐old woman with fever, confusion, and upper extremity superficial venous thrombosis was admitted. Despite screening for infectious, neurologic, and autoimmune causes, no diagnosis was established. Generalized lymphadenopathy was found afterwards. An excised lymph node biopsy with immunohistochemical analysis revealed aggressive B‐cell lymphoma.

## Key Clinical Message

An aggressive B‐cell lymphoma can appear initially with infectious, neurological, or thrombotic consequences. If persistent systemic symptoms and idiopathic bilateral superficial venous thrombosis of the upper extremities are present, clinicians should be prompted to investigate for a potential hematological malignancy to ensure accurate diagnosis and appropriate therapy.

## Graphical abstract

Schematic representation of the disease progression, diagnostic workup, treatment course, and final clinical outcome.

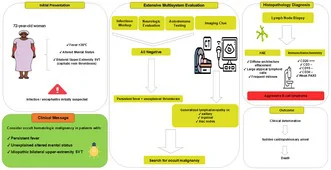

## 1 Introduction

Superficial vein thrombosis (SVT) is less investigated than deep vein thrombosis (DVT) and occurs mainly in the lower extremities. Unusual, bilateral superficial thromboses of the upper extremities represent approximately 15% of upper limb cases [1, 2]. Studies have demonstrated active cancer in 7 to 17% of patients with SVT and thus screening for occult malignancies is important in the absence of obvious local triggers such as intravenous therapy or trauma [3, 4, 5]. Venous thromboembolism (VTE) is also a recognized complication in 5%–10% of patients with lymphoma [6].

Altered mental status (AMS) in older patients not only raises the possibility of paraneoplastic conditions in the differential diagnosis but may also result from more common infectious, metabolic, or neurological etiologies [7, 8]. On the other hand, the aggressive non‐Hodgkin lymphomas known as high‐grade B‐cell lymphomas (HGBCLs), which include diffuse large B‐cell lymphoma (DLBCL) and related conditions [9, 10], can cause a wide spectrum of systemic symptoms due to cytokine dysregulation, hypercoagulability, and, on uncommon occasions, metabolic or paraneoplastic neurological complications [11, 12]. Malignant lymphomas are among the most common neoplastic causes of fever of unknown origin (FUO) [13], and misdiagnosis is common [14].

## 2 Case Presentation

An Iranian woman, aged 72, underwent a sudden alteration in mental status and was admitted to the emergency department on March 2, 2025. Her relatives reported that in the hours leading up to her presentation, she exhibited increasing confusion, disorientation, and fatigue. They reported a temperature exceeding 38°C the previous day and episodes of vomiting.

She was awake and responsive but disoriented to time and place. Vital signs revealed mild tachycardia and mildly decreased oxygen saturation. Neurological examination revealed no focal deficits. Pupillary reactions and extraocular movements were normal, without nystagmus or gaze deviation. There were no fasciculations, muscle atrophy, or involuntary movements; tongue movements were normal, and the gag reflex was intact. There was no nuchal rigidity, and Kernig's and Brudzinski's signs were negative. Speech was notable for dysarthria without aphasia. Cardiovascular and abdominal examinations were unremarkable. Laboratory tests showed high levels of inflammatory markers, leukocytosis, thrombocytosis, and coagulopathy (Table 1). Supplementary specialized immunologic, microbiologic, and imaging investigations were performed and are summarized in Table 2.

**TABLE 1:** Basic laboratory findings during hospitalization.

Category	Findings
Hematology	WBC 12,800–13,700/μL (neutrophils 90%–95%); band forms 9%; metamyelocytes 2%; Hb 8.6–10.1 g/dL; MCV 74–77 fL; MCH 22–24 pg.; MCHC 30–31 g/dL; anisocytosis (+); platelets 510–687 × 10^3^/μL
Coagulation	PT 13–31 s; PTT 14–78 s; INR 4.5 (day 1) → < 1.5 afterward; D‐dimer 1760 ng/mL (day 2)
Metabolic panel	Na 135–144 mEq/L; K 3.2–4.5 mEq/L; BUN/Cr 16/0.8 → 24/0.9; glucose 98–168 mg/dL
Inflammatory markers	ESR 120–130 mm/h; CRP 35 mg/L
Liver function tests	AST 41–106 U/L; ALT 15–26 U/L; ALP 195–320 U/L; bilirubin 0.6–0.7 mg/dL; albumin 2.3–3.7 g/dL
LDH	1001–1504 U/L
Urinalysis	Initial UA normal → repeat (day 6): pyuria (20–22 WBC/hpf), hematuria (25–30 RBC/hpf), yeast present

Abbreviations: ALP, alkaline phosphatase; ALT, alanine aminotransferase; AST, aspartate aminotransferase; BUN, blood urea nitrogen; Cr, creatinine; CRP, C‐reactive protein; ESR, erythrocyte sedimentation rate; Hb, hemoglobin; hpf, high‐power field; INR, international normalized ratio; K, potassium; LDH, lactate dehydrogenase; MCH, mean corpuscular hemoglobin; MCHC, mean corpuscular hemoglobin concentration; MCV, mean corpuscular volume; Na, sodium; PT, prothrombin time; PTT, partial thromboplastin time; UA, urinalysis; WBC, white blood cell count.

**TABLE 2:** Specialized immunologic, microbiologic, and diagnostic studies.

Category	Findings
Microbiology	Urine culture: Yeast positive (day 6); other urine cultures negative; blood cultures negative on days 1, 6, and catheter culture negative (day 11)
Infectious serologies	HBsAg –, HBsAb –, HCV Ab –, HIV Ab –; influenza PCR –, COVID‐19 PCR —
Autoimmune panel	ANA 0.2 IU/mL; anti‐dsDNA 15 IU/mL; RF 8.6 IU/mL; C3 121 mg/dL; C4 31 mg/dL; CH50 120 U/mL
Antiphospholipid profile	Anticardiolipin IgM < 3.0 MPL; IgG 8 GPL; β2‐glycoprotein I IgM 1.5 U/mL; IgG 8 U/mL; lupus anticoagulant 55 s
Other immunologic tests	Anti‐SSA (Ro) 5.7 U/mL (borderline); anti‐SSB (La) < 3 U/mL
Coombs/brucella tests	Direct Coombs –, indirect Coombs –; 2‐mercaptoethanol –; Wright agglutination —
CSF analysis (Day 9)	Glucose 61 mg/dL; protein 9.48 mg/dL; LDH 27 U/L; WBC 5/μL; RBC 0–1/μL; CSF culture negative
Protein electrophoresis (Serum)	Total protein 6 g/dL; albumin 1.8; α1 0.6; α2 1.0; β1 0.4; β2 0.7; γ 1.5 g/dL; no monoclonal spike
Protein electrophoresis (Urine)	Albumin 71.4%; transferrin 2%; RBP 9.3 mg/L; free light chains 26.9 mg/L; α1‐microglobulin 11.1 mg/L; IgG 5.9%; lysozyme 12%; IgA 1.8%; haptoglobulins 9.8%
Blood gas analysis	pH 7.45 (respiratory alkalosis) → pH 7.06, PCO_2_ 49 (acute respiratory acidosis)
Echocardiography	EF ≈50%; mild global hypokinesia; mild MR/TR; calcified AV without stenosis; no vegetations

Abbreviations: ANA, antinuclear antibody; AV, aortic valve; C3/C4, complement components 3 and 4; CH50, total complement activity; CSF, cerebrospinal fluid; EF, ejection fraction; HBsAb, hepatitis B surface antibody; HBsAg, hepatitis B surface antigen; HCV Ab, hepatitis C virus antibody; HIV Ab, human immunodeficiency virus antibody; IgM/IgG, immunoglobulin M/G; MR, mitral regurgitation; PCR, polymerase chain reaction; RBP, retinol‐binding protein; RF, rheumatoid factor; RV, right ventricle; TR, tricuspid regurgitation; VBG, venous blood gas.

The patient was admitted to the intensive care unit with a provisional diagnosis of encephalitis or sepsis of unknown origin. Despite receiving initial assistance, her fever and AMS persisted, necessitating further testing.

### 2.1 Patient History

The patient had a history of Type 2 diabetes mellitus, hypertension, hyperlipidemia, and atrial fibrillation, managed with metformin, rivaroxaban, bisoprolol, valsartan/amlodipine, and atorvastatin. Ten weeks before admission, she developed right groin cellulitis accompanied by transient AMS; neurologic evaluation, including EEG and brain CT, was unremarkable, and symptoms improved with antibiotics. Family history was noncontributory.

### 2.2 Hospital Course and Multisystem Evaluation

Given concern for bacterial meningitis or viral encephalitis, empirical meropenem, vancomycin, and acyclovir were initiated. Cardiovascular, abdominal, and initial neurologic examinations were otherwise unremarkable. On the first hospital day, Doppler ultrasonography of the upper limbs detected thrombosis of both cephalic veins, while evaluation of the lower extremities showed no evidence of deep venous thrombosis. Home rivaroxaban had already been discontinued on admission because of an elevated INR (4.5); once the INR normalized to 1.5 after three days, prophylactic subcutaneous heparin (5000 IU twice daily) was initiated and subsequently transitioned, on cardiology recommendation, to therapeutic enoxaparin (60 mg subcutaneously twice daily).

**FIGURE 1:**
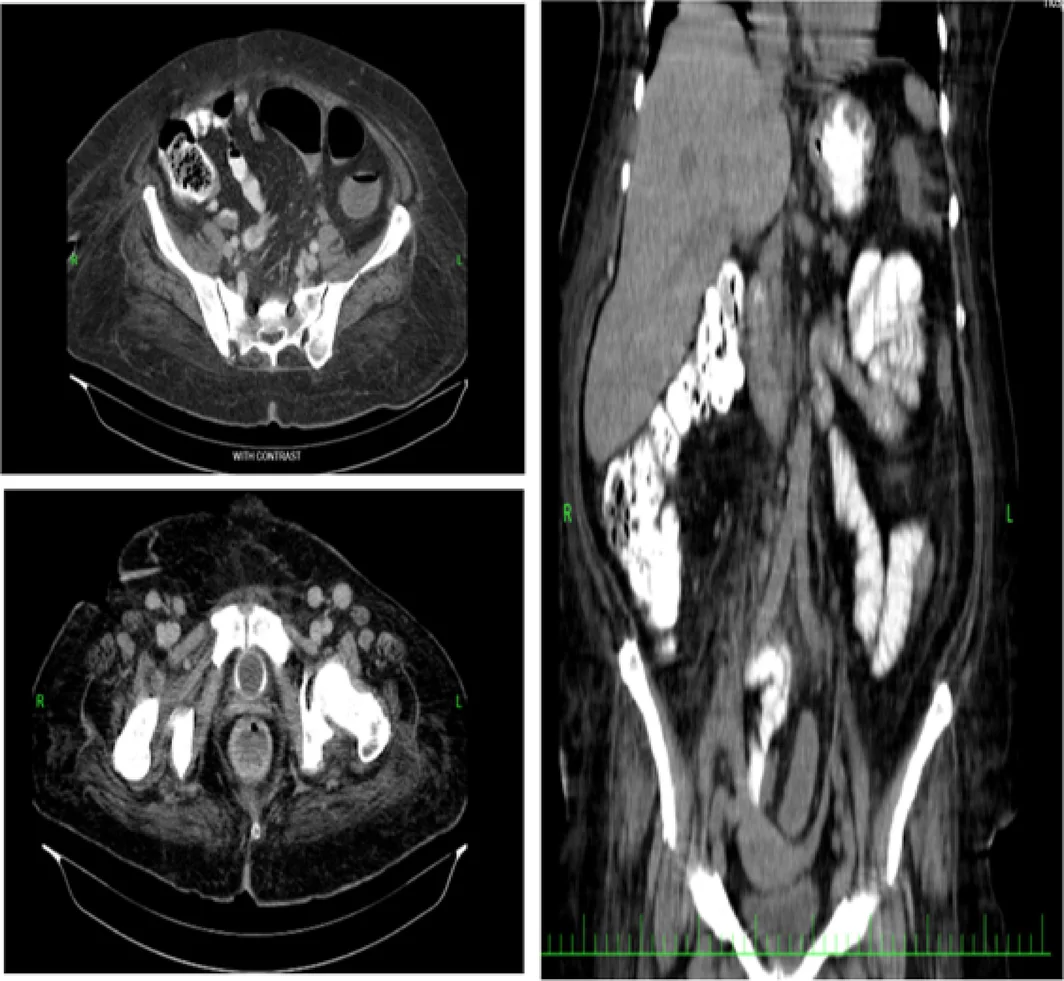
Axial and coronal contrast‐enhanced abdominopelvic CT demonstrating enlarged external iliac lymph nodes (arrows) with surrounding fat stranding, consistent with lymphadenopathy and possible inflammatory or infiltrative involvement.

**FIGURE 2:**
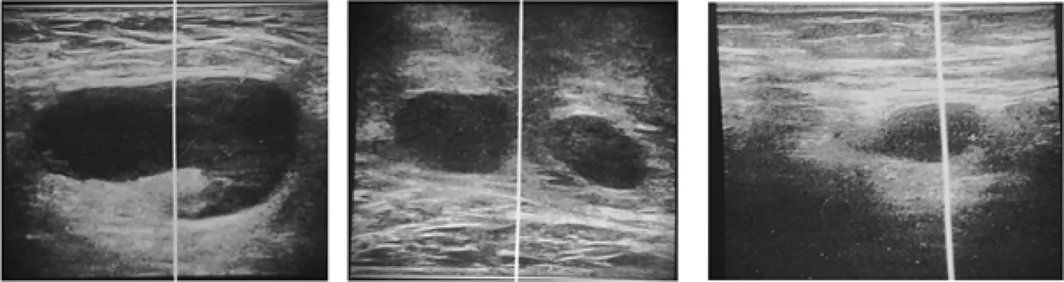
Ultrasound of the axillary region showing multiple enlarged lymph nodes with loss of the normal echogenic fatty hilum. Nodes demonstrated oval morphology, abnormal internal echotexture, and short‐axis diameters of 10 mm (right) and 21 mm (left), findings consistent with pathological lymphadenopathy suggestive of a lymphoproliferative/neoplastic process.

Further cardiac workup did not support infective endocarditis. Electrocardiography demonstrated sinus tachycardia, and transthoracic echocardiography showed preserved left ventricular systolic function (EF approximately 50%), mild valvular insufficiency, and absence of vegetations. A contrast‐enhanced abdominopelvic CT scan obtained on the second day of admission identified enlarged lymph nodes in the pelvic, external iliac, and bilateral inguinal regions (Figure 1). Subsequent physical examination confirmed palpable, firm, painless axillary and inguinal lymphadenopathy, raising suspicion for disseminated nodal disease. Breast ultrasonography revealed bilateral axillary lymphadenopathy with loss of preserved fatty hila, the largest node measuring 21 mm, without detectable breast parenchymal lesions; the examination was categorized as BI‐RADS 0 (Figure 2). Additional microbiologic, immunologic, and diagnostic investigations are summarized in Table 2. Neurologic evaluation revealed AMS with impaired speech but no focal deficits. Therapeutic enoxaparin was held prior to lumbar puncture, performed on Day 9, which showed unremarkable cerebrospinal fluid findings (Table 2). Brain MRI demonstrated mild cortical atrophy and periventricular white‐matter changes without acute intracranial pathology or leptomeningeal enhancement. Because of fluctuating mental status, levetiracetam was initiated empirically for possible subclinical seizure activity. By hospital Day 11, fever and acute confusion improved, allowing transfer to the general ward, although mild temporal disorientation and short‐term memory impairment persisted. On Day 12, an excisional biopsy of a left inguinal lymph node was performed to investigate the generalized lymphadenopathy. On Day 13, given the prolonged interruption of oral anticoagulation, cardiology consultation recommended resuming oral anticoagulation with apixaban (5 mg twice daily); however, the patient experienced sudden cardiopulmonary arrest on Day 13 and could not be revived despite advanced life‐support measures. The specific cause of death was not determined, and the family refused an autopsy.

### 2.3 Histopathology and Immunohistochemistry

**FIGURE 3:**
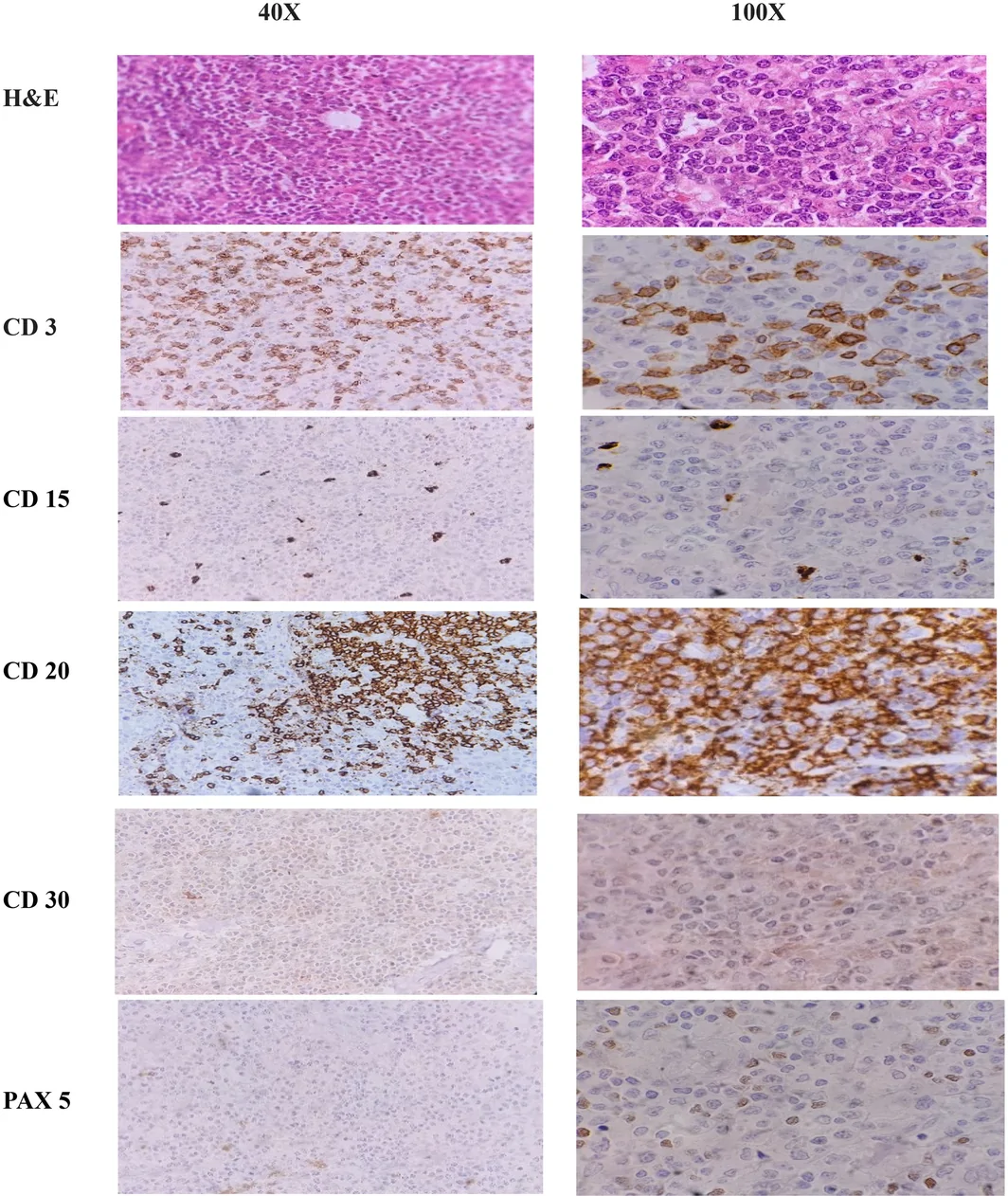
Histopathology and immunohistochemistry of the left inguinal lymph node biopsy.

A diffuse sheet of large atypical lymphoid cells completely effaced normal lymph node architecture without residual follicles in Figure 3 H&E sections from the left inguinal lymph node biopsy. Vesicular nuclei, prominent nucleoli, and frequent mitotic and apoptotic figures indicated a high‐grade lymphoid neoplasm. Immunohistochemistry revealed strong diffuse CD20 positivity in almost 70%–80% of tumor cells, while CD3 stained only background T cells and was negative in tumor cells [15]. CD30 and CD15 were negative. PAX5 showed unexpectedly weak, focal positivity in < 20% of cells. Ki‐67 was not available. The findings are consistent with aggressive B‐cell lymphoma. However, low PAX5 expression, lack of Ki‐67, and lack of MYC/BCL2/BCL6 FISH studies add some diagnostic uncertainty.

## 3 Discussion

The patient's initial presentation—fever, AMS, and bilateral upper‐extremity superficial venous thrombosis—closely mimicked infectious, neurologic, and autoimmune disorders, delaying recognition of the underlying hematologic malignancy. Although generalized lymphadenopathy was identified during admission, the diagnostic workup initially remained focused on acute neurologic and thrombotic manifestations before aggressive B‐cell lymphoma was ultimately established.

Lymphoma is a recognized cause of FUO, particularly in the absence of identifiable infection [16]. In retrospect, persistent fever despite broad‐spectrum antibiotics, negative cultures, and markedly elevated inflammatory markers and lactate dehydrogenase favored an aggressive lymphoproliferative process. Venous thromboembolism is common in lymphoma, including DLBCL, but bilateral superficial venous thrombosis of the upper extremities as a presenting manifestation is unusual [17, 18]. The patient also demonstrated coagulation abnormalities, including prolonged PT/aPTT and elevated D‐dimer. Although rivaroxaban may interfere with coagulation assays, the overall pattern raised concern for malignancy‐associated coagulopathy, possibly reflecting a compensated or low‐grade consumptive process described in aggressive B‐cell lymphomas [19, 20]. Because fibrinogen was unavailable, formal ISTH disseminated intravascular coagulation (DIC) scoring could not be applied, and thrombocytosis argued against overt DIC.

Neuroimaging and cerebrospinal fluid studies showed no structural, infectious, or leptomeningeal explanation for the patient's AMS. The encephalopathy was likely multifactorial, potentially related to infectious or metabolic stress in an elderly patient, with possible contribution from lymphoma‐associated functional CNS effects. Emerging evidence suggests extracranial DLBCL may induce metabolic brain alterations even without structural CNS involvement [21, 22]. Although a paraneoplastic mechanism remained a consideration, it could not be definitively confirmed, as the patient did not undergo a comprehensive assessment according to the revised 2021 Graus diagnostic framework for paraneoplastic neurologic syndromes [23].

Microscopic evaluation revealed marked disruption of normal nodal structure by diffuse proliferation of large atypical lymphoid cells, compatible with an aggressive high‐grade lymphoma. The neoplastic cells showed an immunophenotypic profile of mature B‐cell origin, with strong CD20 expression and negative staining for CD3, CD15, and CD30. However, the diagnostic interpretation should be viewed in light of several limitations. Unexpectedly, PAX5 was weakly and focally expressed in < 20% of the cells, leading to ambiguity in the immunophenotypic interpretation. No formal assessment of proliferative activity was performed on Ki‐67 staining. In addition, MYC, BCL2, and BCL6 molecular studies were not performed, precluding WHO 5th‐edition subclassification between DLBCL‐NOS and high‐grade B‐cell lymphoma with double−/triple‐hit genetics [24, 25]. Consequently, the diagnosis of aggressive B‐cell lymphoma, provisionally most consistent with DLBCL‐NOS, relied on morphology and limited immunohistochemistry. This case illustrates the practical diagnostic challenges of lymphoma classification in resource‐limited settings and reinforces recommendations for referral when advanced molecular testing is unavailable [26, 27].

The patient's sudden cardiac arrest on the 13th day of hospitalization resulted in a fatal outcome. Given the hypercoagulable state associated with malignancy and the patient's prior thrombotic event, pulmonary thromboembolism was considered the most likely cause [28, 29]. However, a definitive diagnosis could not be established because neither confirmatory imaging nor autopsy findings were available. Various probable causes, such as lethal arrhythmia, septic cardiovascular collapse, or metabolic abnormalities, could not be excluded.

## 4 Conclusion

Aggressive B‐cell lymphoma may be difficult to diagnose as initial symptoms can be confused with infections or neurologic or thrombotic events. The underlying malignancy was preceded by fever, AMS, and unexplained superficial bilateral upper‐extremity thrombosis. When patients have persistent systemic symptoms and a negative workup for infectious, autoimmune, and neurologic etiologies, clinicians should have a high index of suspicion for hematologic malignancy. The knowledge of this possibility in advance will help in early diagnosis and management.

## Author Contributions

**Hasan Maghsoudifar:** data curation, software, resources, writing – original draft. **Milad Rezvani:** writing – original draft, writing – review and editing, project administration, investigation, formal analysis, resources, data curation. **Sepehr Zamani:** software, visualization, writing – original draft, writing – review and editing. **Mahdi Naeiji:** writing – original draft, data curation, resources. **Maryam Yarmohammadi:** supervision, methodology, investigation, validation, formal analysis, writing – review and editing. **Maryam Valikhani:** supervision, methodology, validation, visualization, formal analysis, conceptualization, investigation, writing – review and editing.

## Funding

This work was supported by 14050015.

## Ethics Statement

Approved by the Ethics Committee of Shahroud University of Medical Sciences and funded by Shahroud University of Medical Sciences (grant no. 14050015).

## Consent

Written informed consent for publication was obtained from the patient's next of kin.

## Conflicts of Interest

The authors declare no conflicts of interest.

## Data Availability

The data that support the findings of this study are available from the corresponding author upon reasonable request.
